# Discovery of new protein families and functions: new challenges in functional metagenomics for biotechnologies and microbial ecology

**DOI:** 10.3389/fmicb.2015.00563

**Published:** 2015-06-05

**Authors:** Lisa Ufarté, Gabrielle Potocki-Veronese, Élisabeth Laville

**Affiliations:** ^1^Université de Toulouse, Institut National des Sciences Appliquées (INSA), Université Paul Sabatier (UPS), Institut National Polytechnique (INP), Laboratoire d’Ingénierie des Systèmes Biologiques et des Procédés (LISBP), Toulouse, France; ^2^INRA - UMR792 Ingénierie des Systèmes Biologiques et des Procédés, Toulouse, France; ^3^CNRS, UMR5504, Toulouse, France

**Keywords:** metagenomics, discovery of new functions, proteins, high throughput screening, microbial ecosystems, microbial ecology, biotechnologies

## Abstract

The rapid expansion of new sequencing technologies has enabled large-scale functional exploration of numerous microbial ecosystems, by establishing catalogs of functional genes and by comparing their prevalence in various microbiota. However, sequence similarity does not necessarily reflect functional conservation, since just a few modifications in a gene sequence can have a strong impact on the activity and the specificity of the corresponding enzyme or the recognition for a sensor. Similarly, some microorganisms harbor certain identified functions yet do not have the expected related genes in their genome. Finally, there are simply too many protein families whose function is not yet known, even though they are highly abundant in certain ecosystems. In this context, the discovery of new protein functions, using either sequence-based or activity-based approaches, is of crucial importance for the discovery of new enzymes and for improving the quality of annotation in public databases. This paper lists and explores the latest advances in this field, along with the challenges to be addressed, particularly where microfluidic technologies are concerned.

## Introduction

The implications of the discovery of new protein functions are numerous, from both cognitive and applicative points of view. Firstly, it improves understanding of how microbial ecosystems function, in order to identify biomarkers and levers that will help optimize the services rendered, regardless of the field of application. Next, the discovery of new enzymes and transporters enables expansion of the catalog of functions available for metabolic pathway engineering and synthetic biology. Finally, the identification and characterization of new protein families, whose functions, three-dimensional structure and catalytic mechanism have never been described, furthers understanding of the protein structure/function relationship. This is an essential prerequisite if we are to draw full benefit from these proteins, both for medical applications (for example, designing specific inhibitors) and for relevant integration into biotechnological processes.

Many reviews have been published on functional metagenomics these last 10 years. Many of them focus on the strategies of library creation and on bio-informatic developments (Di Bella et al., 2013; Ladoukakis et al., 2014), while others describe the various approaches set up to discover novel targets [like therapeutic molecules (Culligan et al., 2014)] for a specific application. In particular several review papers have been written on the numerous activity-based metagenomics studies carried out to find new enzymes for biotechnological applications, without necessarily finding new functions or new protein families (Ferrer et al., 2009; Steele et al., 2009). The present review focuses on all the functional metagenomics approaches, sequence- or activity-based, allowing the discovery of new functions and families from the uncultured fraction of microbial ecosystems, and makes a recent overview on the advances of microfluidics for ultra-fast microbial screening of metagenomes.

## Sampling Strategies

The literature describes a wide variety of microbial environments sampled in the search for new enzymes. A large number of studies look at ecosystems with high taxonomic and functional diversity, such as soils or natural aquatic environments that are either undisturbed or exposed to various pollutants (Gilbert et al., 2008; Brennerova et al., 2009; Zanaroli et al., 2010). Extreme environments enable the discovery of enzymes that are naturally adapted to the constraints of certain industrial processes, such as glycoside hydrolases and halotolerant esterases (Ferrer et al., 2005; LeCleir et al., 2007), thermostable lipases (Tirawongsaroj et al., 2008), or even psychrophilic DNA-polymerases (Simon et al., 2009). Other microbial ecosystems, such as anaerobic digesters including both human and/or animal intestinal microbiota and industrial remediation reactors, are naturally specialized in metabolizing certain substrates. These are ideal targets for research into particular functions, such as the degrading activity of lignocellulosic plant biomass (Warnecke et al., 2007; Tasse et al., 2010; Hess et al., 2011; Bastien et al., 2013) or dioxygenases for the degradation of aromatic compounds (Suenaga et al., 2007).

Some studies refer to enrichment steps that occur before sampling, with the aim of increasing the relative abundance of micro-organisms that have the target function. This enrichment can be done by modifying the physical and chemical conditions of the natural environment (van Elsas et al., 2008) or by incorporating the substrate to be metabolized *in vivo* (Hess et al., 2011) or *in vitro*, in reactors (DeAngelis et al., 2010) or mesocosms (Jacquiod et al., 2013). Through stable isotopic probing and cloning of the DNA of micro-organisms able to metabolize a specifically labeled substrate for the creation of metagenome libraries, it is possible to increase the frequency of positive clones by several orders of magnitude (Chen and Murrell, 2010). These approaches require functional and taxonomic controls at the different stages of enrichment, which are often sequential, to prevent the proliferation of populations dependent on the activity of the populations preferred at the outset. These kinds of checks are difficult to do *in vivo*, where there would actually be an increased risk of selecting populations able to metabolize only the degradation products of the initial substrate, to the detriment of those able to attack the more resistant original substrate with its more complex structure.

## Functional Screening: New Challenges for the Discovery of Functions

**FIGURE 1:**
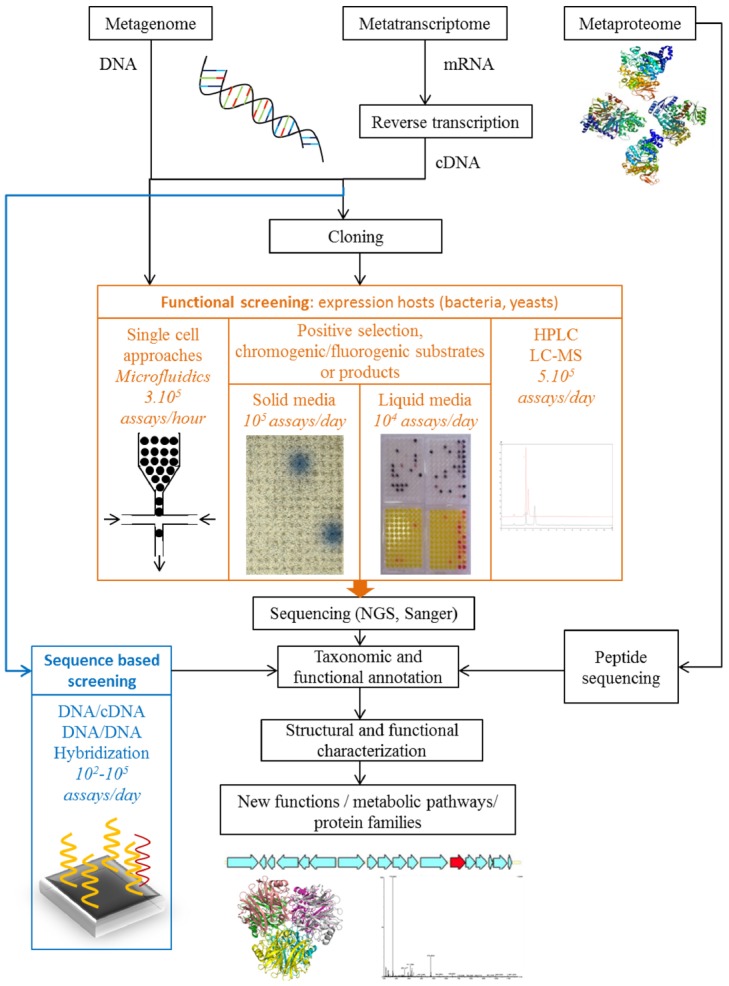
**Strategies for the functional exploration of metagenomes, metatranscriptomes and metaproteomics to discover new functions and protein families**.

Two complementary approaches can be used to discover new functions and protein families within microbial communities. The first involves the analysis of nucleotide, ribonucleotide or protein sequences, and the other the direct screening of functions before sequencing (Figure 1).

### The Sequence, Marker of Originality

There have been a number of large-scale random metagenome sequencing projects (Yooseph et al., 2007; Vogel et al., 2009; Gilbert et al., 2010; Qin et al., 2010; Hess et al., 2011) over the past few years, resulting in catalogs listing millions of genes from different ecosystems, the majority of which are recorded in the GOLD^1^ (RRID:nif-0000-02918), MG-RAST^2^ (RRID:OMICS_01456) and EMBL-EBI^3^ (RRID:nlx_72386) metagenomics databases. At the same time, the obstacles inherent to metatranscriptomic sampling (fragility of mRNA, difficulty with extraction from natural environments, separation of other types of RNA) have been removed, opening a window into the functional dynamics of ecosystems according to biotic or abiotic constraints (Saleh-Lakha et al., 2005; Warnecke and Hess, 2009; Schmieder et al., 2012). Metatranscriptomes sequencing has thus enabled the identification of new gene families, such as those found in microbial communities (prokaryotes and/or eukaryotes) expressed specifically in response to variations in the environment (Bailly et al., 2007; Frias-Lopez et al., 2008; Gilbert et al., 2008) and new enzyme sequences belonging to known carbohydrate active enzymes families (Poretsky et al., 2005; Tartar et al., 2009; Damon et al., 2012).

Regardless of the origin of the sequences (DNA or cDNA, with or without prior cloning in an expression host), the advances made with automatic annotation, most notably thanks to the IMG-M (RRID:nif-0000-03010) and MG-RAST (RRID:OMICS_01456) servers (Markowitz et al., 2007; Meyer et al., 2008), now make it possible to quantify and compare the abundance of the main functional families in the target ecosystems (Thomas et al., 2012), identified through comparison of sequences with the general functional databases: KEGG (RRID:nif-0000-21234) (Kanehisa and Goto, 2000), eggNOG (RRID:nif-0000-02789) (Muller et al., 2010), and COG/KOG (RRID:nif-0000-21313) (Tatusov et al., 2003). They also enable research into specific protein families, thanks to motif detection using Pfam (RRID:nlx_72111) (Finn et al., 2010), TIGRFAM (RRID:nif-0000-03560) (Selengut et al., 2007), CDD (RRID:nif-0000-02647) (Marchler-Bauer et al., 2009), Prosite (RRID:nif-0000-03351) (Sigrist et al., 2010), and HMM model construction (*Hidden Markov Models*; Söding, 2005). Other servers can be used to interrogate databases specialized in specific enzymatic families (Table 1).

**TABLE 1:** **Examples of databases specialized in enzymatic functions of biotechnological interest**.

**Databases**	**Enzymes**	**References**
MetaBioME	Enzymes of industrial interest	Sharma et al. (2010)
CAZy	carbohydrate active enzymes	Cantarel et al. (2012)
(RRID:OMICS_01677)	Auxiliary redox enzymes for lignocellulose degradation	Levasseur et al. (2013)
CAT	carbohydrate active enzymes	Park et al. (2010)
(RRID:OMICS_01676)		
LccED	Laccases	Sirim et al. (2011)
LED	Lipases	Pleiss et al. (2000)
(RRID:nif-0000-03084)		
MEROPS	Proteases	Rawlings et al. (2012)
(RRID:nif-0000-03112)		
ThYme	Thioesterases	Cantu et al. (2011)

Finally, the performance of methods used to assemble next generation sequencing reads is set to open up access to a plethora of complete genes to feed expert databases, which currently only contain a tiny percentage of genes from uncultivated organisms—less than 1% for the CAZy database (RRID:OMICS_01677), for example—while the majority of metagenomic studies published target ecosystems with a high number of plant polysaccharide degradation activities by carbohydrate active enzymes (André et al., 2014).

Even based on a large majority of truncated genes, metagenomes and metatranscriptomes functional annotation enables *in silico* estimations of the functional diversity of the ecosystem and identification of the most original sequences within a known protein family. It is then possible to use PCR (Polymerase Chain Reaction) to capture those sequences specifically, and test their function experimentally to assess their applicative value. In this way, the sequencing of the rumen metagenome (268 Gb) enabled identification of 27,755 coding genes for carbohydrate active enzymes, and isolation of 51 active enzymes belonging to known families specifically involved in lignocellulose degradation (Hess et al., 2011).

PCR, and more generally DNA/DNA or DNA/cDNA hybridization, also make it possible to directly capture coding genes for protein families that are abundant and/or expressed in the target ecosystem, but with no need for *a priori* large-scale sequencing. This strategy requires the conception of nucleic acid probes or PCR primers using consensus sequences specific to known protein families. There are plenty of examples of the discovery of enzymes in metagenomes using these approaches, for instance bacterial laccases (Ausec et al., 2011), dioxygenases (Zaprasis et al., 2009), nitrites reductases (Bartossek et al., 2010), hydrogenases (Schmidt et al., 2010), hydrazine oxidoreductases (Li et al., 2010), or chitinases (Hjort et al., 2010) from various ecosystems. The Gene-Targeted-metagenomics approach (Iwai et al., 2009) combines PCR screening and amplicon pyrosequencing to generate primers in an iterative manner and increase the structural diversity of the target protein families, for example the dioxygenases from the microbiota of contaminated soil. Elsewhere, the use of high-density functional microarrays considerably multiplies the number of probes and is therefore a low-cost way of obtaining a snapshot of the abundance and diversity of sequences within specific protein families and even, where the DNA or cDNA has been cloned (He et al., 2010; Weckx et al., 2010), directly capturing targets of interest while rationalizing sequencing. Using a similar strategy, the solution hybrid selection method enables the selection of fragments of coding DNA for specific enzymatic families using 31-mers capture probes. Applied to the capture of cDNA, this method provides access to entire genes which can be then cloned and their activity tested (Bragalini et al., 2014). Solution hybrid selection can therefore be used to explore the taxonomic and functional diversity of all protein families. More especially, this approach opens the way for the selection and characterization of families that are highly represented in a microbiome but whose function remains unknown, in order to further the understanding of ecosystemic functions and discover novel biocatalysts.

Metaproteomics has recently proved its worth in identifying new protein families and/or functions. Paired with genomic, metagenomic and metatranscriptomic data (Erickson et al., 2012), it provides access to excellent biomarkers of the functional state of the ecosystem. Recent developments, such as high-throughput electrospray ionization paired with mass spectrometry, enable full metaproteome analysis after separation of proteins by liquid chromatography. It is thus possible to highlight hundreds of proteins with no associated function and new enzyme families playing a key functional role in the ecosystem (Ram et al., 2005).

This latter example illustrates the need for research and/or experimental proof of function for proteins where the function remains unknown (products of orphan genes or, on the contrary, genes highly prevalent in the microbial realm but that have never been characterized) or poorly annotated. In fact, annotation errors, which are especially common for multi-modular proteins such as carbohydrate active enzymes, are spread at an increasing rate as a result of the explosion in the number of functional genomics and meta-genomic, -transcriptomic and -proteomic projects. New annotation strategies, most notably based on the prediction of the three-dimensional structure of proteins, are also worth exploring (Uchiyama and Miyazaki, 2009). However, at the present time, it is very difficult to predict the specificity of substrate and the mechanism of action (and therefore the function of the protein) on the basis of sequence or even structure, especially where there is no homologue characterized from a structural and functional point of view. Functional screening can address this challenge.

### Activity Screening: Speeding up the Discovery of Biotechnology Tools

There are three prerequisites for this approach: (i) the cloning of DNA or cDNA in an expression vector for the creation of, respectively, metagenomic or metatranscriptomic libraries, (ii) heterologous expression of cloned genes in a microbial host, iii) the conception of efficient phenotypic screens to isolate the clones of interest that produce the target activity, also referred to as “hits.”

Using this approach, the functions of a protein can be accessed without any prior information on its sequence. It is therefore the only way of identifying novel protein families that have known functions or previously unseen functions (as long as an adequate screen can be developed). Finally, it helps to rationalize sequencing efforts and focus them only on the hits: for example, those that are of biotechnological interest. The expression potential of the selected heterologous host, the size of the DNA inserts and the type of vectors all determine the success of functional screening. Short fragments of metagenomic DNA (smaller than 15 kb, and most often between 2 and 5 kb), or cDNA for the metatranscriptomic libraries, cloned in plasmids under the influence of a strong expression promoter, enable the overexpression of a single protein, and the easy recovery and sequencing of the hits’ DNA (Uchiyama and Miyazaki, 2009). On the other hand, fragments of bacterial DNA measuring between 15 and 40 kb, 25 and 45 kb or even 100 and 200 kb, cloned respectively in cosmids, fosmids or bacterial artificial chromosomes, can be used to explore a functional diversity of several Gb per library and, above all, provide access to operon-type multigene clusters, coding for complete catabolic or anabolic pathways This is of major interest for the discovery of cocktails of synergistic activities that degrade complex substrates such as plant cell walls for biorefineries. This strategy also ensures high reliability for the taxonomic annotation of inserts, and can even be used to identify the mobile elements responsible for the plasticity of the bacterial metagenome, mediated by horizontal gene transfers (Tasse et al., 2010). However, it requires sensitive activity screens, since the target genes are only weakly expressed, controlled by their own native promoters.

*Escherichia coli*, whose transformation efficiency is exceptionally high, even for fosmids or bacterial artificial chromosomes, remains the host of choice in the immense majority of studies published. The first exhaustive functional screening study of a fosmid library revealed that *E. coli* can be used to express genes from bacteria that are very different from a taxonomical point of view, including a large number of Bacteroidetes and Gram-positive bacteria (Tasse et al., 2010), contrary to what had been predicted by *in silico* detection of expression signals compatible with *E. coli* (Gabor et al., 2004). However, the value of developing shuttle vectors to screen metagenomic libraries in hosts with different expression and secretion potentials, for example *Bacillus*, *Sphingomonas*, *Streptomyces*, *Thermus*, or the α-, β- and γ–proteobacteria (Taupp et al., 2011; Ekkers et al., 2012) must not be underestimated, if we are to unlock the functional potential of varied taxons and increase the sensitivity of screens. Finally, it is still very difficult to get access to the uncultivated fraction of eukaryotic microorganisms, due to the lack of screening hosts with sufficient transformation efficiency for the creation of large clone libraries (and thus the exploration of a vast array of sequences) and compatible with the post-translational modifications required to obtain functional recombinant proteins from eukaryotes. Thus, at the present time, only a few studies have been published on the enzyme activity-based screening of metatranscriptomic libraries (making it possible to do away with introns) of eukaryotes from soil, rumen and the gut of the termite (Bailly et al., 2007; Findley et al., 2011, Sethi et al., 2013).

Regardless of the type of library screened, the functional exploration of hundreds of thousands of clones is required, whereas the hit rate rarely exceeds 6‰ (Duan et al., 2009; Bastien et al., 2013). This requires very high throughput primary screens, in a solid medium before or after the automated organization of libraries in 96- or 384-well micro-plate format, in a liquid medium after enzymatic cell lysis and/or thawing and freezing (Bao et al., 2011), or using UV-inducible auto-lytic vectors (Li et al., 2007). This stage is very often followed by medium or low throughput characterization of the properties of the hits obtained, particularly to assess their biotechnological interest (Tasse et al., 2010).

Two generic strategies, used at throughputs exceeding 400,000 tests per week, have been and continue to be applied widely. Positive selection on a medium containing, for example, substrates to be metabolized as the sole source of carbon, can be used to isolate enzymes (Henne et al., 1999), complete catabolic pathways (Cecchini et al., 2013), or membrane transporters (Majerník et al., 2001). This approach also helps easily identify antibiotic resistant genes (Diaz-Torres et al., 2006). The use of chromogenic (Beloqui et al., 2010; Bastien et al., 2013; Nyyssönen et al., 2013), fluorescent (LeCleir et al., 2007), or opalescent substrates or reagents, such as insoluble polymers or proteins (Mayumi et al., 2008; Waschkowitz et al., 2009), or simply the observation of an original clone phenotype, has already enabled the isolation of several 100 catabolic enzymes, like the numerous hydrolases of very varied taxonomic origin (Simon and Daniel, 2009), some of which were coded by genes that are very abundant in the target ecosystem (Jones et al., 2008; Gloux et al., 2011), but also, although much less frequently, new oxidoreductases (Knietsch et al., 2003). Novel enzymes (laccases, esterases and oxygenases in particular) from microbial communities of very diverse origins (soil, water, activated sludge, digestive tracts) have been highlighted for their capacity to degrade pollutants such as nitriles (Robertson and Steer, 2004), lindane (Boubakri et al., 2006), styrene (Van Hellemond et al., 2007), naphthalene (Ono et al., 2007), aliphatic and aromatic carbohydrates (Uchiyama et al., 2004; Brennerova et al., 2009; Lu et al., 2012), organophosphorus (Kambiranda et al., 2009; Math et al., 2010), or plastic materials (Mayumi et al., 2008).

The discovery of proteins involved in prokaryote-eukaryote interactions (Lakhdari et al., 2010) or anabolic pathways is rarer, since it often requires the development of complex screens and lower throughputs. Nonetheless, a few examples of simple screens, based on the aptitude of metagenomic clones to inhibit the growth of a strain by producing antibacterial activity or to complement an auxotrophic strain for a specific compound, have enabled the identification of new pathways for the synthesis of antimicrobials (Brady and Clardy, 2004) or biotin (Entcheva et al., 2001). Nano-technologies, and in particular the latest developments focused on the medium-throughput screening of libraries obtained by combinatorial protein engineering, enable the design of custom microarrays and covered with one to several 100 specific enzymatic substrates, the processing of which may be followed by fluorescence, chemiluminescence, immunodetection, surface plasmon resonance or mass spectrometry (André et al., 2014). Nanostructure-initiator mass spectrometry technology, combining fluorescence and mass spectrometry, is the first example of a functional metagenomic application for the discovery of anabolic enzymes, namely sialyltransferases (Northen et al., 2008).

### The Immense Challenges of Ultra-fast Screening (Figure 2)

**FIGURE 2:**
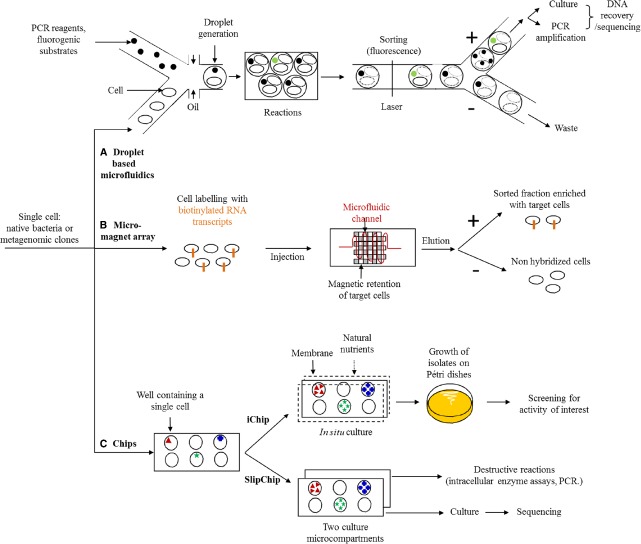
**Microfluidic strategies for new enzyme screening. (A)** Droplet based microfluidics: single cells are encapsulated with probes or fluorogenic substrates to create microdroplets, where reactions happen (substrate degradation, PCR). The hits are sorted using fluorescence detection. Non-lysed cells are cultured and DNA fragments from lysed cells are amplified. Both methods allow the recovery and sequencing of DNA. **(B)** Micro-magnet array: target cells are labeled with biotinylated RNA transcripts probes and injected inside the microchannel. Target cells are captured in the channel thanks to magnetic forces while non-targets cells pass through the device. **(C)** Chips: the chip wells are filled with a single cell. The iChip is covered by membranes, and reintroduced into original environment, where natural nutrients flow through membranes. Colonies are further isolated on Petri dishes to be screened for the activity of interest. The SlipChip is composed of two culture microcompartments which are further separated for destructive and non-destructive assays.

Microfluidic technologies are of undeniable interest when it comes to reaching screening rates of a million clones per day. The substrate induced gene-expression screening method has been developed to use fluorescence-activated cell sorting to isolate plasmidic clones containing genes (or fragments of genes) that induce the expression of a fluorescent marker in response to a specific substrate. However, this technique is only suited to small substrates that are non-lethal and internalizable for the host strain (Uchiyama and Watanabe, 2008). Finally, the advances made over the past few years in cellular compartmentalization (Nawy, 2013), selective sorting, based on sequence detection (Pivetal et al., 2014; Lim et al., 2015) or specific metabolites (Kürsten et al., 2014) and the control of reaction kinetics (Mazutis et al., 2009) in microfluidic circuits should allow for a huge acceleration in the discovery of new proteins and metabolic pathways expressed in prokaryotes and eukaryotes in an intercellular, membrane or extracellular manner.

The very first examples of metagenome functional exploration applications have already been used to establish the proof of concept regarding the effectiveness of microfluidics in the discovery of new bioactive molecules and new enzymes. For example, droplet-based microfluidics technology was recently used by the teams of A. Griffiths and A. Drevelle to isolate new strains producing cellobiohydrolase and cellulase activities at a rate of 300,000 cells sorted per hour, using just a few microliters of reagent, i.e., 250,000 times less than with the conventional technologies mentioned above (Najah et al., 2014). Here, soil bacteria and a fluorescent substrate were co-encapsulated in micro-droplets in order to sort cells on the basis of the extracellular activity only. In fact, the strategy used, which requires the seeding of cells on a defined medium after sorting, is not compatible with the detection of intracellular enzymes, which require a lethal lysis step to convert the substrate. Applying a similar principle, the ultra-rapid sorting of eukaryote cells encapsulated with their substrate now also makes it possible to select yeast clones presenting extracellular enzymatic activities (Sjostrom et al., 2014). This technology should, in the short term, make it possible to explore the functional diversity of uncultivated eukaryotes at a very high throughput, by directly sorting fungal populations or libraries of metatranscriptomic clones. In the latter case, access to the sequence involved in the target activity will be easy, since the libraries are built using hosts whose culture is well managed, with insertion of the metatranscriptomic cDNA fragment into a specific region of the genome. Where sorting is done without cloning of the metagenome or metatranscriptome, only microorganisms capable of growth on a defined medium can be recovered, which hugely limits access to functional diversity.

To increase the proportion of cultivable organisms, Kim Lewis’ team recently used the iChip to simultaneously isolate and cultivate soil bacteria thanks to the delivery of nutrients from the original medium, into which the iChip is introduced, via semi-permeable membranes. This method enables an increase in cultivable organisms ranging from 1 to 50%. Using colonies cultivated in the chip, the clones isolated in a Petri dish were screened for the production of antimicrobial compounds (Ling et al., 2015). A novel antibiotic was thus identified, together with its biosynthesis pathway, after sequencing and functional annotation of the complete genome.

It is quite another matter when it comes to selecting, on the basis of intracellular activity, completely uncultivable organisms or metagenomic clones containing DNA inserts of several dozen kbp, which are difficult to amplify using PCR. In this case, to liberate the enzymes in question, we are required to include a cellular lysis step, preventing seeding after sorting. On the other hand, this approach is compatible with the sorting of plasmid clone libraries, where the metagenomic or metatranscriptomic inserts can easily be amplified using PCR, on the basis of just a few dozen lysed cells. For libraries with large DNA inserts, the barriers are now being broken down, most notably thanks to the development of the SlipChips microfluidic approach (Ma et al., 2014), which uses two culture microcompartments, where the content of one can be lysed for the detection of enzymatic activities, for example, and the other is used as a backup replicate for the culture and recovery of subsequent DNA for sequencing. In spite of these recent, highly encouraging developments, the proof of concept has not yet been established for the identification of new functions and intracellular metabolic pathways.

## Conclusion

The rapid expansion of meta-omic technologies over the past decade has shed light on the functions of the uncultivated fraction of microbial ecosystems. A huge number of enzymes have been discovered, in particular through experimental approaches to functional metagenomes exploration. Where their performance can be rapidly assessed within the framework of a known process, or where they catalyze new, previously undescribed reactions, many of them have provided new tools for industrial biotechnologies. However, several challenges still need to be addressed to speed up the rate at which new functions are discovered and to make optimal use of the functional diversity that so far remains unexplored. Firstly, while the uncultivated prokaryote fraction of microbial communities is still extensively studied, the functions of the eukaryote fraction are relatively unexplored from an experimental angle, even though they play a fundamental role for numerous ecosystems. Secondly, in the majority of cases, the functions discovered using meta-omic approaches play a catabolic role, mainly involved in the deconstruction of plant biomass or in bioremediation. It is thus necessary to develop functional screens to access anabolic functions and enrich the catalog of reactions available for synthetic biology. Finally, there are very few studies aimed at identifying the role of protein families that are highly prevalent in the target ecosystem but that have not yet been characterized, even though some of them could be considered as biomarkers of the functional state of the microbial community. Indeed, sequence-based functional metagenomic projects continuously highlight many sequences annotated as domains of unknown function in the Pfam database (RRID: nlx_72111) (Bateman et al., 2010; Finn et al., 2014), some with 3D structures solved thanks to structural genomics initiatives, and available in the Protein Data Bank (RRID: nif-0000-00135). With the goal of characterizing these new protein families and identifying previously unseen functions from the selection the most prevalent protein families (those containing the highest number of homologous sequences without any associated function) in the target ecosystem, the integration of structural, biochemical, genomic and meta-omic data is now also possible (Ladevèze et al., 2013). It allows to benefit from the huge amount of long scaffolds now available in sequence databases, and to access the genomic context of the targeted genes in order to facilitate functional assignation. In the next few years, these strategies should enhance our understanding of how microbial ecosystems function and, at the same time, enable greater control over them.

## Author Contributions

LU, GPV, EL contributed equally to this work.

### Conflict of Interest Statement

The authors declare that the research was conducted in the absence of any commercial or financial relationships that could be construed as a potential conflict of interest.

## Footnotes

1 http://www.genomesonline.org/cgi-bin/GOLD/index

2 http://metagenomics.anl.gov/

3 http://www.ebi.ac.uk/metagenomics
